# Supplementary material for: Impact of agile management on project performance: Evidence from I.T sector of Pakistan
Source: PLoS One. 2021 Apr 5;16(4):e0249311. doi: 10.1371/journal.pone.0249311 (PMC8021201; doi:10.1371/journal.pone.0249311)
Supplement: S1 Appendix — (DOCX) [file pone.0249311.s001.docx]

**S1 Appendix.**

**Survey Form**

**Part A: Demographics**

| **Age** | [ 1] 20-30 Yrs. | [ 2] 30-40 Yrs. |
| --- | --- | --- |
|  | [ 3] 40-50 Yrs. | [ 4] 50 Yrs. and Above |
|  |  |  |
| **Tenure** | [ 1] Less than 1 Year | [ 2] 1 – 2 Years |
|  | [ 3] 2 – 5 Years | [ 4] 5 – 10 Years |
|  | [ 5] 10 Years & Above |  |
|  |  |  |
| **Employment Sector** | [ 1] Public | [ 2] Private |
|  |  |  |
| **Employment Status** | [ 1] Permanent | [ 2] Contractual |
| **Employment City** |  | |

**Part B: Organizational Variables**

|  |  | Strongly Disagree | Disagree | Neutral | Agree | Strongly Agree |
| --- | --- | --- | --- | --- | --- | --- |
| **Part B: Agile Management Practices** | | | | | | |
| 1 | Your organization has the ability to challenge and outperform new entries in the market. | -1- | -2- | -3- | -4- | -5- |
| 2 | Your organization has the ability to predict the trend of service and/or product life cycle. | -1- | -2- | -3- | -4- | -5- |
| 3 | Your organization has the ability to maintain its position among its direct competitors in the local market. | -1- | -2- | -3- | -4- | -5- |
| 4 | Your organization has the ability to maintain its position among its direct competitors in global market. | -1- | -2- | -3- | -4- | -5- |
| 5 | Your organization has the ability to predict its market share, considering the intensity of competition. | -1- | -2- | -3- | -4- | -5- |
| 6 | Your organization has a strategic basis for competition (competition on: price, product differentiation, time, quality, service). | -1- | -2- | -3- | -4- | -5- |
| 7 | Your organization has the ability to operate efficiently at different levels of output. | -1- | -2- | -3- | -4- | -5- |
| 8 | Your organization has the ability to effectively increase or decrease aggregate services and/or production in response to customers. | -1- | -2- | -3- | -4- | -5- |
| 9 | Your organization can maintain performance standards when producing a wide variety of services and/or products. | -1- | -2- | -3- | -4- | -5- |
| 10 | Your organization can produce different service and/or product types without major changeover. | -1- | -2- | -3- | -4- | -5- |
| 11 | Your organization can produce, simultaneously or periodically, multiple services and/or products in an operating cycle. | -1- | -2- | -3- | -4- | -5- |
| 12 | Employees in your organization can perform different types of operations effectively. | -1- | -2- | -3- | -4- | -5- |
| 13 | Employees in your organization can be transferred easily between organization units/ departments. | -1- | -2- | -3- | -4- | -5- |
| 14 | Your organization has the ability to effectively respond to changes in planned delivery times. | -1- | -2- | -3- | -4- | -5- |
| 15 | Your organization can changeover quickly from one service and/or product to another. | -1- | -2- | -3- | -4- | -5- |
| 16 | Your organization can quickly discover changes in customer preferences. | -1- | -2- | -3- | -4- | -5- |
| 17 | Your organization makes quick decisions in reaction to price change. | -1- | -2- | -3- | -4- | -5- |
| 18 | Your organization usually makes regular interdepartmental meetings in reaction to external changes. | -1- | -2- | -3- | -4- | -5- |
| 19 | Your organization responds quickly to competitors’ campaigns. | -1- | -2- | -3- | -4- | -5- |
| 20 | Interdepartmental activities are well coordinated in your organization. | -1- | -2- | -3- | -4- | -5- |
| **Part C: Project Complexity** | | | | | | |
| 21 | The project teams at your organizations are deployed cross-functionally. | -1- | -2- | -3- | -4- | -5- |
| 22 | The projects at your organization, involve multiple external contractors and vendors. | -1- | -2- | -3- | -4- | -5- |
| 23 | The project at your organization, involve coordinating multiple user units. | -1- | -2- | -3- | -4- | -5- |
| 24 | The system at your organization, involves real-time data processing. | -1- | -2- | -3- | -4- | -5- |
| 25 | The projects at your organization, involve multiple software environments. | -1- | -2- | -3- | -4- | -5- |
| 26 | The end-users related to your projects, often observe changes in their organizational structure. | -1- | -2- | -3- | -4- | -5- |
| 27 | The end-users related to your projects, often observe changes in their business process. | -1- | -2- | -3- | -4- | -5- |
| 28 | Implementing the undertaken project often causes changes in the end-user’s organizational structure. | -1- | -2- | -3- | -4- | -5- |
| 29 | Implementing the undertaken projects often cause changes in the end-user’s business processes. | -1- | -2- | -3- | -4- | -5- |
| 30 | In regard to the projects at your organization, the end-user’s information needs often change rapidly. | -1- | -2- | -3- | -4- | -5- |
| 31 | In regard to the projects at your organization, IT architecture that the project depended on often change rapidly. | -1- | -2- | -3- | -4- | -5- |
| 32 | In regard to the projects at your organization, IT infrastructure that the project depended on change rapidly. | -1- | -2- | -3- | -4- | -5- |
| **Part E: Leadership Competencies** | | | | | | |
| 33 | Your supervisor acts in an ethical manner. | -1- | -2- | -3- | -4- | -5- |
| 34 | Your supervisor displays consistency between words and actions. | -1- | -2- | -3- | -4- | -5- |
| 35 | Your supervisor protects confidential information. | -1- | -2- | -3- | -4- | -5- |
| 36 | Your supervisor effectively handles multiple demands and competing priorities. | -1- | -2- | -3- | -4- | -5- |
| 37 | Your supervisor spends time on the most important issues, not just the most urgent ones. | -1- | -2- | -3- | -4- | -5- |
| 38 | Your supervisor seeks feedback from others. | -1- | -2- | -3- | -4- | -5- |
| 39 | Your supervisor adjusts behavior in, response to feed­back and experience. | -1- | -2- | -3- | -4- | -5- |
| 40 | Your supervisor demonstrates awareness of own strengths and weaknesses. | -1- | -2- | -3- | -4- | -5- |
| 41 | Your supervisor works constructively under stress and pressure. | -1- | -2- | -3- | -4- | -5- |
| 42 | Your supervisor views problems as opportunities and mistakes as progress. | -1- | -2- | -3- | -4- | -5- |
| **Part G: Project Performance** | | | | | | |
| 43 | Your organization, deliver projects within targeted schedule. | -1- | -2- | -3- | -4- | -5- |
| 44 | Your organization, deliver projects within targeted budget. | -1- | -2- | -3- | -4- | -5- |
| 45 | Your organization, deliver projects within targeted efforts. | -1- | -2- | -3- | -4- | -5- |
| 46 | Your organization, maintains an environment with high work moral. | -1- | -2- | -3- | -4- | -5- |
| 47 | Your organization deliver projects, fulfilling the quality criterions. | -1- | -2- | -3- | -4- | -5- |
| 48 | Your organization deliver projects with which clients are satisfied. | -1- | -2- | -3- | -4- | -5- |
